# Motivation and competence of participants in a learner-centered student-run clinic: an exploratory pilot study

**DOI:** 10.1186/s12909-017-0856-9

**Published:** 2017-01-25

**Authors:** Tim Schutte, Jelle Tichelaar, Ramon S. Dekker, Abel Thijs, Theo P. G. M. de Vries, Rashmi A. Kusurkar, Milan C. Richir, Michiel A. van Agtmael

**Affiliations:** 1 Department of Internal Medicine, Pharmacotherapy Section, VU University Medical Center, De Boelelaan 1117 - room ZH 4A50, 1081 HZ Amsterdam, The Netherlands; 2 RECIPE (Research & Expertise Center In Pharmacotherapy Education), Amsterdam, The Netherlands; 3 Department of Internal Medicine, VU University Medical Center, Amsterdam, The Netherlands; 4 School of Medical Sciences, VU University Medical Center, Amsterdam, The Netherlands

**Keywords:** Student-run clinic, Motivation, Medical education, Pharmacotherapy

## Abstract

**Background:**

The Learner-Centered Student-run Clinic (LC-SRC) was designed to teach and train prescribing skills grounded in a real-life context, to provide students with early clinical experience and responsibility. The current studies’ theoretical framework was based on the Self-determination Theory. According to the Self-determination Theory, early involvement in clinical practice combined with a high level of responsibility makes the LC-SRC an environment that can stimulate intrinsic motivation. We investigated the different types of motivation and the proficiency in CanMEDS competencies of the participating students.

**Method:**

Type of motivation was measured using the Academic Motivation Scale and Intrinsic Motivation Inventory. CanMEDS competencies were evaluated by faculty using a mini-clinical examination and by the students themselves using a post-participation questionnaire.

**Results:**

The 29 participating students were highly intrinsic motivated for this project on all subscales of the Intrinsic Motivation Inventory. Motivation for medical school on the Academic Motivation Scale was high before and was not significantly changed after participation. Students considered that their CanMEDS competencies “Collaborator”, “Communicator”, “Academic”, and “Medical expert” had improved. Their actual clinical team competence was judged by faculty to be at a junior doctor level.

**Conclusion:**

Students showed a high level of intrinsic motivation to participate in the LC-SRC and perceived an improvement in competence. Furthermore their actual clinical competence was at junior doctor level in all CanMEDS competencies. The stimulating characteristics of the LC-SRC, the high levels of intrinsic motivation and the qualitative comments of the students in this study makes the LC-SRC an attractive place for learning.

**Electronic supplementary material:**

The online version of this article (doi:10.1186/s12909-017-0856-9) contains supplementary material, which is available to authorized users.

## Background

In the undergraduate medical curricula, there is a need of opportunities for students to practice prescribing [1]. Prescribing encompasses a range of activities from performing a consultation, identifying a need for drug therapy, selecting and prescribing the appropriate drug to being involved in the subsequent management of the patient [1, 2]. *McLellan* et al. suggest that the way to teach and provide training in prescribing skills is to design interventions grounded in a real-life context, so that students can be observed and evaluated in the context of their (future) workplace [3]. Learning in the (future) workplace (Workplace Learning) is “as old as medicine itself” however does not necessarily contain specific responsibilities for students [4]. Giving students a feeling of responsibility for patient care makes their clinical experiences more ‘real’ and legitimate, and might stimulate student motivation [5]. Such enrichment of responsibility (for patient care) is thought to be an important factor to improve the training of rational prescribing skills of medical students [6]. This combination of context/workplace learning, early clinical experience, and sense of responsibility has been described as learning by doing [7].

Based on this concept, a Learner-Centered Student-run Clinic (LC-SRC) was started at the VUmc School of Medical Sciences in 2013 [8, 9]. The LC-SRC is a learner-centered project, as opposed to regular SRCs that primarily focus on providing (free) care [7]. In the LC-SRC students get the opportunity to train themselves in complex competencies such as patient communication, therapeutic reasoning, and prescribing in a real context. The LC-SRC concept and development is based on the conceptual framework of learning by doing, as an example within the more general experiential learning theory by Kolb [7, 9, 10]. Besides the experience itself, its timing and the attending responsibilities of the clinical experience are important. Experiences should be real and legitimate for optimal learning effects and involvement [4, 5, 11].

**Fig. 1:**
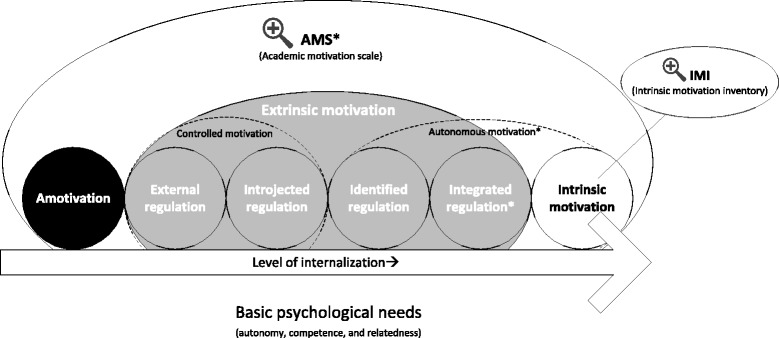
The theoretical self-determination continuum (from left, Amotivation (least autonomous) to right, Intrinsic motivation (most autonomous), and the position of the different motivational sub-types of motivation as measured with the AMS (e.g. Amotivation, External Regulation, Introjected Regulation and Identified Regulation). *Integrated Regulation is not measured in/with the AMS questionnaire and is therefore no part of the calculated Autonomous motivation subscale. The IMI was used to study intrinsic motivation in depth, see Table 1

Student motivation is in general a neglected aspect in the designing of medical curricula [12]. In spite of description of the best principles for doing this [13], few initiatives consider and measure the effect of interventions catered to enhance student motivation in medical education [14], especially so in undergraduate medical curricula. The theoretical framework for this study is based on the Self-determination Theory (SDT). According to this theory, motivation can be classified into intrinsic and extrinsic types; the intrinsic motivation originates from within oneself, and extrinsic originates from external factors [15–17]. An example of intrinsic motivation is to learn to prescribe out of genuine interest and the desire to help one’s patients; an example of extrinsic motivation is to learn about certain drugs for an exam one has to pass. Intrinsic motivation depends on the fulfilment of three basic psychological needs, namely, autonomy, competence, and relatedness [15]. It is considered the best form of motivation to promote in-depth learning and to improve performance and competence in learning outcomes [16, 18–20]. Based on SDT, an individual is never thought to be exclusive intrinsically or extrinsically motivated and motivation is different for different activities. Furthermore, both intrinsic and extrinsic types of motivation are always present in differing levels, which can be influenced. The key feature in the transformation of extrinsic to intrinsic motivation is internalization. The level of internalization differs across the SDT continuum (see Fig. 1) [21]. Internalization itself is stimulated by similar components that stimulate intrinsic motivation, being autonomy, competence, and relatedness [22]. The higher the level of internalization, the more autonomous is the motivation. Autonomous motivation is calculated as an average of the scores on score identified regulation and intrinsic motivation, whereas controlled motivation is calculated as an average of the introjected regulation and external regulation scores (see Fig. 1).

An early involvement in clinical practice combined with a high level of responsibility makes the LC-SRC an environment that can fulfil all three basic psychological needs for students intrinsic motivation and internalization: autonomy by giving students responsibility of patients, competence through feedback from supervisors and confidence in handling patients and relatedness through working in teams of peers, near-peers and supervisors [18]. Therefore we hypothesize that participation in LC-SRC will stimulate the intrinsic and autonomous motivation of students for this type of (pharmacotherapy) teaching and learning [12, 15–17, 23].

Since the LC-SRC was designed to stimulate intrinsic and autonomous motivation, and thereby the competence of students, our research questions were:

1. What type of motivation do students have for this educational innovation?
2. Does the motivation for medical education change after participation in this innovation?
3. How does this innovation influence students’ proficiency in CanMEDS competencies?

## Methods

### Setting

The regular VUmc curriculum (6-years) consists of 3 years of preclinical education (bachelor degree), followed by 3 years of clinical education (master/medical degree). This study was performed within the extracurricular LC-SRC project of the VU University Medical Center, Amsterdam [8, 9]. The LC-SRC project for 1^st^ to 6^th^ year students focuses on early clinical experience with responsibility for real patients. Compared to regular clerkships, students are in the lead in the LC-SRC, they are the principal contacts for “their” patients, and are responsible for patient care including follow up. A key feature of this patient care is the proposition of a reasoned and customized (pharmacotherapy) treatment plan [9].

The students work in teams and are jointly responsible for outpatient consultations within the Department of Internal Medicine, with real patients (who have medical insurance), under supervision of an internist. Teams changed during the course of the study, based on the availability of individual students. The student teams prepare their consultations, based on the (electronic) medical record, and are encouraged to read more about the medical conditions of “their” patients. In addition, students can attend interdisciplinary discussions (e.g. radiology and microbiology), and consult nurses, administrative personnel, and medical doctors from other disciplines. Each student has a specific role and responsibility during the consultation. The third-year student leads the team, performs the consultation, and is coached by the fifth-year student. The first-year student complements with questions and makes annotations for the medical record. Patients are requested to visit the LC-SRC for follow-up after their first (regular) consultation with a resident. If appropriate, follow-up consultations to monitor treatment are planned, whenever possible, with the same student teams, to stimulate longitudinal learning. The consultations take place in the outpatient clinic of our hospital. After each consultation, the supervising internist provides students with feedback on their performance.

### Participants

Participation in the LC-SRC project is extracurricular and voluntary, students were invited to apply before/during a regular lecture by sending a letter regarding their expectations and their experiences in healthcare. All students who participated in the LC-SRC pilot from March to July 2013 (*n* = 31) were invited to take part in the present research project. They were sent an e-survey before and after their participation. All mini-Clinical Evaluation Exercises (mini-CEX) of consultations in the LC-SRC, within this period, were included.

### Measurements - motivation

Motivation was assessed with two standardized validated questionnaires, the Academic Motivation Scale (AMS) [24–26] and the Intrinsic Motivation Inventory (IMI) [27–29]. The AMS was used to differentiate between intrinsic and extrinsic motivation for studying at the medical school [24]. Intrinsic motivation for this particular project was measured using the IMI-subscales Interest, Usefulness, and Perceived Choice (Likert scale of 1–7). The Interest/Enjoyment subscale is considered the primary self-report measure of intrinsic motivation [30]. Furthermore, together the IMI-subscales are related to the three psychological needs, which are all important components within intrinsic motivation, and moreover to stimulate integration/regulation of extrinsic motivation into autonomous motivation [15, 22, 27, 28, 30]. The AMS was completed twice, pre and post participation. The IMI was completed once, post participation. Written feedback was collected by means of open questions in the post-participation questionnaire.

### Measurements - competencies

**Fig. 2:**
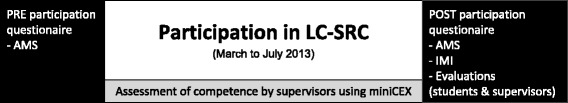
Measurements of motivation and competence in time during the study. The pre participation questionnaire consisted of the baseline characteristics and the AMS questionnaire as shown in Table 1. The post participation questionnaire was longer and consisted of the AMS and the IMI (both provided in Table 2) and evaluation questions. These evaluation questions encompassed the students’ perceived improvement on their competencies (Fig. 4) and their reflections regarding the LC-SRC (Table 3)

The CanMEDS competencies framework [31] was chosen to evaluate clinical competencies, as both students and supervisors are used to the competences described within this framework, from their experience within the regular medical curriculum. A post-participation questionnaire was used to evaluate students’ perceived improvement in CanMEDS competencies. In order to objectively measure students’ CanMEDS competencies, these were evaluated by faculty from internal medicine, using mini-CEX, to grade and provide feedback for student teams after each consultation. These CanMEDS mini-CEX were regularly used by faculty to evaluate clinical competence of students in their regular clerkships. The seven CanMEDS competencies were scored on a 5-point Likert scale (compared to the level of a junior doctor, 3 meant achieving a junior doctor level, >3 better, <3 worse) (See Additional file 1, CanMEDS mini-CEX). Supervisors were questioned about their opinion on the improvement in medical knowledge, communication, clinical reasoning, and pharmacotherapeutic knowledge/skills of students after their participation in the LC-SRC. Figure 2 indicates which tests, questionnaires and assessments were performed at which time point.

### Analysis

Quantitative data were imported in SPSS (IBM, version 20.0). The AMS subscale scores were calculated (see Table 1). Autonomous motivation was calculated as the mean scores of intrinsic motivation and identified regulation subscales of the AMS. Controlled motivation was calculated as the mean of introjected regulation and external regulation [32] (see Table 1 and Fig. 1). Reliability was assessed with Cronbach’s alpha. Differences between motivation in subgroups (male vs. female and preclinical vs. clinical students), were analysed with the non-parametric Mann–Whitney-U-test given the likely non-normal distribution in these small samples. The difference between motivation before and after participation, measured with AMS (ordinal variables), was analysed with a student’s paired t-test. This parametric test was considered feasible given its high resolution (it could be considered as interval data as most subscales range from 4 to the maximum of 28), and the use of parametric tests for 5- or 7-point Likert scales [33, 34]. Interpretation of statistical significance is based on a Bonferroni correction for multiple testing. Descriptive statistics were used to report mini-CEX outcomes and student-perceived improvement in CanMEDS competencies. Pearson correlations were calculated for all motivational measures (IMI and post-participation AMS subscales) and the student-perceived improvement in CanMEDS competencies. The qualitative data (open questions, written feedback and comments) were analysed using content analysis [35]. Two authors (TS and RD) read and interpreted the students’ feedback and comments simultaneously and resolved differences through discussion and consensus. Identified themes were discussed and agreed upon within the full research team.

**Table 1:** Motivation questionnaires used in this study, items indicated with (R) are reverse scored items

Academic Motivation Scale (AMS)			
The AMS, Academic Motivation Scale, was originally described by Vallerand et al. [26] in French as the EME (*l’échelle de motivation en education*) [26], and in 1992 in English as the AMS [24]. This instrument has 28 items, scored on a 7-point Likert scale. The individual items are grouped into subscales for which the scores are calculated as the average score of the individual items within these subscales (these subscales are Intrinsic, extrinsic identified, Extrinsic introjected, Extrinsic external regulation, Amotivation, and the more overarching subscales controlled motivation and autonomous motivation, see also Fig. 1). The AMS is based on the conceptual framework of the Self-determination Theory and is used to differentiate between intrinsic and extrinsic motivation. In 1993 Vallerand et al. studied the validity and reliability of the AMS to measure motivation (types) [25].			
Using the scale (1–7), indicate to what extent each of the following items presently corresponds to one of the reasons why you go to medical school 1: Does not correspond at all 4: Corresponds moderately 7: Corresponds exactly	1	Because with only a medical school degree I would not find a high-paying job later on.	Extrinsic – external regulation
	2	Because I experience pleasure and satisfaction while learning new things.	Intrinsic – to know
	3	Because I think that a medical school education will help me better prepare for the career I have chosen.	Extrinsic – identified regulation
	4	For the intense feelings I experience when I am communicating my own ideas to others.	Intrinsic – experience stimulation
	5	Honestly, I don’t know; I really feel that I am wasting my time in medical school.	Amotivation
	6	For the pleasure I experience while surpassing myself in my medical studies.	Intrinsic – towards accomplishment
	7	To prove to myself that I am capable of completing my medical degree.	Extrinsic – introjected regulation
	8	In order to obtain a more prestigious job later on.	Extrinsic – external regulation
	9	For the pleasure I experience when I discover new things never seen before.	Intrinsic – to know
	10	Because eventually it will enable me to enter the job market in a field (medical) that I like.	Extrinsic – identified regulation
	11	For the pleasure that I experience when I read interesting medical authors.	Intrinsic – experience stimulation
	12	I once had good reasons for going to medical school; however, now I wonder whether I should continue.	Amotivation
	13	For the pleasure that I experience while I am surpassing myself in one of my personal accomplishments.	Intrinsic – towards accomplishment
	14	Because of the fact that when I succeed in medical school I feel important.	Extrinsic – introjected regulation
	15	Because I want to have “the good life” later on.	Extrinsic – external regulation
	16	For the pleasure that I experience in broadening my knowledge about medical subjects which appeal to me.	Intrinsic – to know
	17	Because this will help me make a better choice regarding my medical career orientation.	Extrinsic – identified regulation
	18	For the pleasure that I experience when I feel completely absorbed by what certain medical authors have written.	Intrinsic – experience stimulation
	19	I can’t see why I go to medical school and frankly, I couldn’t care less.	Amotivation
	20	For the satisfaction I feel when I am in the process of accomplishing difficult academic activities.	Intrinsic – towards accomplishment
	21	To show myself that I am an intelligent person.	Extrinsic – introjected regulation
	22	In order to have a better salary later on.	Extrinsic – external regulation
	23	Because my medical studies allow me to continue to learn about many things that interest me.	Intrinsic – to know
	24	Because I believe that a few additional years of education (medical) will improve my competence as a worker.	Extrinsic – identified regulation
	25	For the “high” feeling that I experience while reading about various interesting medical subjects.	Intrinsic – experience stimulation
	26	I don’t know; I can’t understand what I am doing in medical school.	Amotivation
	27	Because medical school allows me to experience a personal satisfaction in my quest for excellence in my studies.	Intrinsic – towards accomplishment
	28	Because I want to show myself that I can succeed in my medical studies.	Extrinsic – introjected regulation
Intrinsic Motivation Inventory (IMI)			
The IMI, Intrinsic motivation inventory, was originally used by Ryan in 1982 to study intrinsic motivation and self-regulation in laboratory experiments [29]. Later on, the IMI has also been used in educational settings (sports, dental education) to study psychometric properties in real practice [28, 36]). The IMI has several subscales, of which *interest/enjoyment* is considered the main self-report measure of intrinsic motivation [30]. The subscale *usefulness* is considered relevant in the process of internalization, that drives the transition from controlled to autonomous motivation (see Fig. 1) [27]. Other subscales include *perceived choice*, *perceived competence*, *effort*, *felt pressure and tension*, and *relatedness*. Intrinsic motivation for this particular project was measured using the IMI-subscales subscale *interest/enjoyment, usefulness* and *perceived choice,* for which the questions are displayed below. Given the IMI is project/subject specific, it could only be measured in participants who participated, and was therefore measured after participation, see Fig. 2.			
Using the scale (1–7), indicate to what extent each of the following items presently corresponds to your opinion 1: Not true at all 4: Somewhat true 7: Very true	1	I believe that doing this project could be of some value for me.	Value/usefulness
	2	I believe I had some choice about doing this project.	Perceived choice
	3	While I was doing this project, I was thinking about how much I enjoyed it.	Interest/enjoyment
	4	I believe that doing this project is useful for improved concentration.	Value/usefulness
	5	This project was fun to do.	Interest/enjoyment
	6	I think this project is important for my improvement.	Value/usefulness
	7	I enjoyed doing this project very much.	Interest/enjoyment
	8	I really did not have a choice about doing this project.	Perceived choice (R)
	9	I did this project because I wanted to.	Perceived choice
	10	I think this is an important project.	Value/usefulness
	11	I felt like I was enjoying the project while I was doing it.	Interest/enjoyment
	12	I thought this was a very boring project.	Interest/enjoyment (R)
	13	It is possible that this project could improve my studying habits.	Value/usefulness
	14	I felt like I had no choice but to do this project.	Perceived choice (R)
	15	I thought this was a very interesting project.	Interest/enjoyment
	16	I am willing to do this project again because I think it is somewhat useful.	Value/usefulness
	17	I would describe this project as very enjoyable.	Interest/enjoyment
	18	I felt like I had to do this project.	Perceived choice (R)
	19	I believe doing this project could be somewhat beneficial for me.	Value/usefulness
	20	I did this project because I had to.	Perceived choice (R)
	21	I believe doing this project could help me do better in medical school.	Value/usefulness
	22	While doing this project I felt like I had a choice.	Perceived choice
	23	I would describe this project as very fun.	Interest/enjoyment
	24	I felt like it was not my own choice to do this project.	Perceived choice (R)
	25	I would be willing to do this project again because it has some value for me.	Value/usefulness

### Ethical considerations

The institutional review board of the VU University Medical Center approved the research proposal, deeming that it did not fall under the scope of the Dutch Medical Research Involving Human Subjects Act (WMO) (ID 2013/364). Nevertheless, all stakeholders were informed about the study in advance, gave oral and/or written consent, and participated on a voluntary basis. Final responsibility for clinical decisions was at the supervisor level. The data were analysed anonymously and at a group level.

## Results

**Fig. 3:**
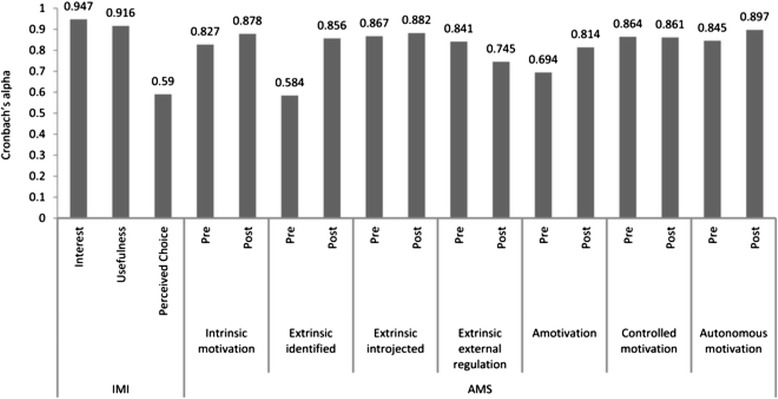
Reliability as tested with Cronbach’s alpha of used motivational scales

During the pilot period between March and July 2013, 31 medical students (11 first-year, 10 third-year, and 10 fifth-year students) performed 31 consultations. The students performed the consultations in teams, and the individual students participated on average for two half-days (range 1–5). Cronbach’s alphas for reliability were 0.95, 0.92, and 0.59 for the IMI Interest, Usefulness, and Perceived Choice subscales, respectively (Fig. 3). The reliability score for the Usefulness subscale was higher than that reported earlier [36]. Cronbach’s alphas for the reliability of the AMS subscale of Controlled Motivation, Autonomous Motivation and Amotivation were 0.86, 0.85, and 0.69, respectively, before participation and 0.86, 0.90, and 0.81, respectively, after participation (Fig. 3). These reliability scores were consistent with those reported earlier [37, 38].

### Participation outcomes on motivation

Twenty-nine students, out of which 25 were females, completed the IMI questionnaire (post-participation) about intrinsic motivation to participate in the LC-SRC (response rate 93.5%). They scored a mean of 6.20 (SD 0.67) on the Interest/Enjoyment subscale. The mean scores for the Usefulness and Perceived Choice subscales were 6.02 (SD 0.81) and 5.93 (SD 0.72), respectively (Table 2). We found no significant differences in the scores of the IMI-subscales between male and female students.

**Table 2:** motivational scores before and after participation in the LC-SRC

	N	Pre score Mean (SD)	Post score Mean (SD)	Statistical significance (2-sided, paired t-test)	Interpretation of Statistical significance after Bonferroni correction for multiple testing
IMI: Interest	29	-	6.20 (0.67)	-	-
IMI: Usefulness	29	-	6.02 (0.81)	-	-
IMI: Perceived Choice	29	-	5.93 (0.72)	-	-
AMS: Intrinsic motivation	25	5.37 (0.69)	5.31 (0.69)	p 0.532	Non-significant
AMS: Extrinsic identified regulation	25	5.92 (0.65)	5.64 (0.90)	p 0.124	Non-significant
AMS: Extrinsic introjected regulation	25	4.19 (1.42)	4.40 (1.27)	p 0.201	Non-significant
AMS: Extrinsic external regula0tion	25	3.77 (1.15)	4.06 (1.12)	p 0.073	Non-significant
AMS: Amotivation	25	1.21 (0.42)	1.20 (0.40)	p 0.788	Non-significant
AMS: Controlled motivation	25	3.98 (1.01)	4.23 (1.03)	p 0.055	Non-significant
AMS: Autonomous motivation	25	5.51 (0.59)	5.39 (0.64)	p 0.217	Non-significant

In 25 paired cases (80,6% of participants), of which 20 were female, motivation was measured with the AMS before and after participation. Intrinsic motivation for attending medical school was 5.37 (SD 0.69) before and 5.31 (SD 0.69) after LC-SRC participation (paired t-test *p* = 0.532). Corresponding before and after scores were 3.98 (SD 1.01) and 4.23 (SD 1.03), respectively, for Controlled Motivation (paired t-test, *p* = 0.055), 5.51 (SD 0.59) and 5.39 (SD 0.64) for Autonomous Motivation (paired t-test, *p* = 0.217), and 1.21 (SD 0.42) and 1.20 (SD 0.40) for Amotivation (paired t-test, *p* = 0.788) (Table 2). We found no significant difference on eventual change in motivation (before-after) within either the subgroups of male and female students or between the pre-clinical students (1^st^ and 3^rd^ year) and already clinical students (5^th^ year).

### Participation and competence

**Fig. 4:**
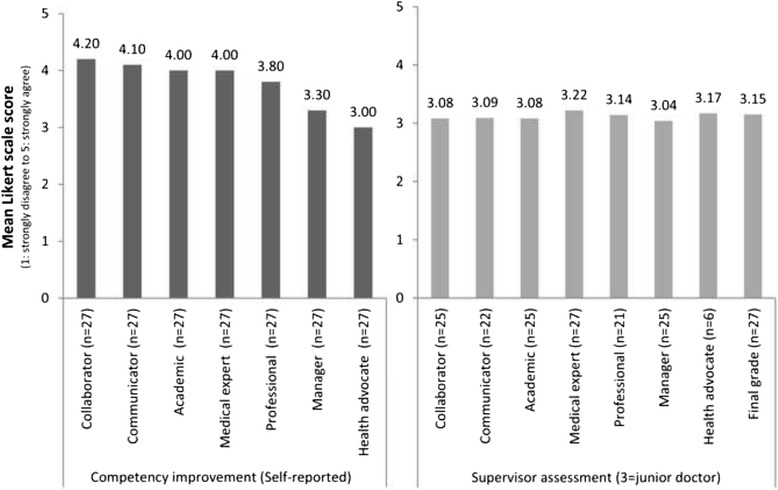
*Left* Self-reported improvement in competence of participating students, scored on a Likert scale 1–5, (strongly disagree to strongly agree), and *Right* CanMEDS competencies of student teams, assessed by supervisors using the MiniCEX (Likert scale 1–5, strongly disagree to strongly agree; score 3 is the level of a junior doctor). Not every CanMEDS role was applicable for every consultation

Twenty-seven students (response rate 87.1%) evaluated whether their CanMEDS competencies had improved (Likert scale 1–5, strongly disagree to strongly agree). Students considered that their proficiency in the CanMEDS competencies of “Collaborator”, “Communicator”, “Academic”, and “Medical expert” had improved after participation (Likert score ≥4.0). Faculty staff evaluated their clinical competence as being at a junior doctor level (Likert score 3.15 (SD 0.60) on mini-CEX at the team level) (Fig. 4). Additionally, in the post-participation questionnaire three out of four supervisors reported that in their opinion the communication skills, medical knowledge, and clinical reasoning of the participating students had improved. The fourth supervisor doubted whether these proficiencies had improved. Half of the supervisors regarded it doubtful that the pharmacotherapeutic knowledge and skills of the students had improved after their participation, and the other two did think the students’ knowledge and skills had improved.

Table 3 shows the responses and comments of student participants, arranged by the categories identified during the analysis, based on the three psychological needs as described in the SDT, i.e. autonomy, competence, and relatedness. Twenty-nine students provided feedback, these responses reflected the students valued working together in teams and enjoyed the supervision (*n* = 14) (relatedness), they valued their roles and opportunities in contributing to real patientcare (*n* = 13) (autonomy), and they felt responsible and competent in the consultations, including the patient management/proposing a treatment plan (*n* = 11) (autonomy and competence).

**Table 3:** Quotations/Motivational statements by participating students (Some quotes fit into more than one theme)

Basic Psychological needs	Quotations/Motivational statements
Autonomy	
Feeling responsible and autonomous regarding real patients	*“Students are given freedom AND responsibility”*
	*“Independence and learning to think for yourself about patient management”*
	*“By doing everything yourself, you learn what is involved in practicing as a medical doctor in an outpatient clinic. For instance filling out the forms and the medical record. That was really instructive!”*
	*“It was really enjoyable to diagnose a patient, and to come up with a therapy or treatment plan, all by yourself”* [also competence]
	*“it’s special to be able to follow a patient, perform the follow-up yourself”*
	*“Motivated fellow students, kind supervisors that are approachable, you are able to ask anything you want to while you are very well stimulated tot think for yourself”*
Competence	
- Feeling to have learned - Feeling to be able to contribute - Affirmation of capabilities (by themselves or others)	*“Being considered of value by patients”*
	*“Being able to come up with a treatment plan”*
	*“it’s special to be able to follow a patient, perform the follow-up yourself”*
	*“It is great to apply what you learned in real practice”*
Relatedness	
- Feeling privileged - Being part of a special group - Getting a chance - Working together	*“Very good opportunity, in the bachelor phase, to practise in a team things like patient contact, medical records and other aspects of a doctor’s work.”*
	*“The collaboration with doctors and fellow students, the fact that you can share your experiences with younger students, so that they learn something from you.”*
	*“Motivated fellow students, kind supervisors that are approachable, you are able to ask anything you want to while you are very well stimulated tot think for yourself”*
	*“Enjoyable atmosphere together with the supervising doctors, within our student team as with the project coordinators”*

### Correlations between motivation and competence improvement

Table 4 shows the Pearson correlations between motivation (subscales of IMI and AMS) with the effect sizes and perceived improvement in CanMEDS competencies. Strong positive correlations (defined as *r* = 0.7 to 0.9) [39] were found between the IMI subscale Interest and the IMI subscale Usefulness (r 0.724; *p* < 0.01), and between improvement in the CanMEDS competencies Communicator and Collaborator (r 0.713; *p* < 0.01). Moderate positive correlations (r 0.4 to 0.6) [39] were found between the IMI subscale Interest and improvement in the CanMEDS competencies Medical Expert and Academic (0.522; *p* < 0.01) (0.656 *p* < 0.01), and between the IMI subscale Usefulness and all self-reported improvement in the CanMEDS competencies except Collaborator (r 0.401 to 0.672). Furthermore, multiple positive correlations (significant, moderate) were found between the perceived improvement in CanMEDS competencies (see Table 4).

**Table 4:** Pearson correlations of IMI subscales, AMS subscales and student-reported competence. Correlations were considered ‘strong positive’ for *r* = 0.7 to 0.9, and ‘moderate positive’ for *r* = 0.4 to 0.6

Pearson Correlations - Motivation and perceived improvement in competences														
		1	2	3	4	5	6	7	8	9	10	11	12	13
1. IMI - Interest	29	-												
2. IMI - Usefulness	29	**0.724** ^**b**^	-											
3. IMI - Perceived choice	29	0.222	**0.438** ^**a**^	-										
4. AMS Intrinsic motivation	27	0.350	**0.420** ^**a**^	**0.518** ^**b**^	-									
5. AMS Controlled motivation	27	0.102	0.197	−0.009	0.053	-								
6. AMS Autonomous motivation	27	0.379	**0.461** ^**a**^	**0.534** ^**b**^	**0.951** ^**b**^	0.250	-							
7. CANM Medical expert	29	**0.522** ^**b**^	**0.601** ^**b**^	0.329	0.226	0.134	0.261	-						
8. CANM Communicator	29	0.361	**0.509** ^**b**^	0.285	0.167	0.098	0.117	**0.612** ^**b**^	-					
9. CANM Collaborator	29	0.355	0.362	0.215	0.076	0.198	0.083	**0.551** ^**b**^	**0.713** ^**b**^	-				
10. CANM Academic	29	**0.656** ^**b**^	**0.672** ^**b**^	0.280	**0.445** ^**a**^	0.195	**0.474** ^**a**^	**0.590** ^**b**^	**0.642** ^**b**^	**0.586** ^**b**^	-			
11. CANM Professional	29	0.364	**0.401** ^**a**^	−0.021	0.358	0.118	0.309	**0.383** ^**a**^	**0.433** ^**a**^	0.256	**0.546** ^**b**^	-		
12. CANM Manager	29	0.333	**0.514** ^**b**^	0.060	0.298	0.287	0.291	**0.429** ^**a**^	0.319	0.189	**0.603** ^**b**^	**0.576** ^**b**^	-	
13. CANM Health advocate	29	0.200	**0.436** ^**a**^	**0.425** ^**a**^	0.186	0.236	0.271	**0.456** ^**a**^	0.189	0.147	**0.384** ^**a**^	0.318	**0.380** ^**a**^	-

Statistical significant correlations are indicated in bold

^a^ Correlation is significant at the 0.05 level (2-tailed)

^b^ Correlation is significant at the 0.01 level (2-tailed)

### Regression analysis

A regression analysis was performed to find out whether IMI-subscales Interest, Usefulness, and Perceived Choice affected Autonomous motivation. We found that the only IMI-subscale with a significant predictive effect was Perceived Choice (*R* ^2^ = 0.309, *p* = 0.018), the effects of Interest (*p* = 0.392) and Usefulness (*p* = 0.708) were not significant.

## Discussion

In this study we investigated the different types of motivation and the proficiency in CanMEDS competencies of the participating students in our Learner-Centered Student-run Clinic (LC-SRC). Type of motivation was measured using the Academic Motivation Scale (AMS, both pre- and post-participation) and Intrinsic Motivation Inventory (IMI, after participation). CanMEDS competencies were evaluated by faculty using a mini-clinical examination and by the students themselves using a post-participation questionnaire.

Students were intrinsically motivated to participate in the LC-SRC project, which was based on the conceptual framework of learning by doing and SDT [16, 17]. While motivation for attending the medical school in general (which was already high) did not change during the study, the students’ intrinsic motivation for the LC-SRC was positively correlated with their perceived improvement in their CanMEDS competencies medical expert and academic. The faculty involved in the assessment considered that the student teams performed at a junior doctor level.

We did not find any studies in medical education that have used the IMI, although the instrument has been used in studies of sports and dental education [28, 36]. Intrinsic motivation in our current study was comparable to the levels measured in dental students who completed a preclinical laboratory course in operative dentistry, they reported IMI subscale scores for Usefulness in two cohorts (5.9 and 6.2) [36]. In experimental manipulations, *rationale*, *acknowledgement,* and *perceived choice* were identified to positively influence internalization and intrinsic motivation [27].

We think that the IMI can prove useful to educationalists and curriculum developers for evaluating intrinsic motivation for medical education (projects), especially because many current educational practices (i.e., lectures and the motivation through pressure such as exams) do not stimulate intrinsic (and autonomous) motivation [18]. This is the first study to explicitly measure (intrinsic) motivation for participation in SRC projects using a validated quantitative method [7, 40–42]. We found intrinsic motivation to be high, especially on the Interest/enjoyment and Usefulness subscales, which we had anticipated given the characteristics of the project [23]. Kusurkar et al. described 12 tips to stimulate intrinsic motivation in medical education, including the stimulation of autonomy (providing optimal challenges), encouraging participation, and encouraging students to accept more responsibility for their learning (and moreover for their patients) [23]. These components formed the basis of the conceptual framework of learning by doing in the LC-SRC [7, 9]. The important role of intrinsic motivation in the LC-SRC design was further confirmed by the open feedback given by the participating students, feedback which was consistent with the basic psychological needs described in the SDT (autonomy, competence, and relatedness) [16, 17]. Students referred to the SRC as an ‘instructive/educational’ place where they could function in relative ‘autonomy’, and as ‘a special opportunity for early clinical teaching and to learn and think independently about (pharmaco)therapy’.

Motivation for medical school in general did not improve, possibly because the duration (time and intensity) of this project was too short to achieve a change in motivation, and the level of motivation was already high before participation in this group. Overall, the level of autonomous motivation (AMS subscale) observed in this study seemed to be higher (pre 5.51 and post 5.39) compared to an earlier study in our institution (male 5.309, female 5.353) [38]. In this same study, the levels of Controlled motivation (male 4.464, female 3.996) were comparable to ours (pre 3.98 and post 4.23) [38]. Although this comparison suggests superior autonomous motivation in LC-SRC participants compared to regular curriculum students, these data must be interpreted with caution.

An alternative explanation for the lack of improvement of motivation is that students found the LC-SRC so exciting and challenging that they were less motivated to attend regular education such as lectures and training with fictional cases. This explanation is supported by the students’ comments, which indicated they were disappointed in their limited role in patient care during their regular clerkships.

We found no significant difference on (change in) motivation between male and female participants on the AMS and IMI subscales. Previous studies did find different motivation profiles in males and females. In these studies males tend to have higher levels of controlled motivation, and lower levels of autonomous motivation and Relative Autonomous Motivation (RAM) [38, 43–45]. A possible explanation for this difference is the majority of female participants in this study and the small sample size. Therefore this study has a limited power to detect gender differences.

The students perceived that their proficiency in CanMEDS competencies improved as a result of LC-SRC participation. Nevertheless, self-reported improvement is known to be biased and is poorly correlated with other performance measures [46]. However, the faculty in this study also reported that the students’ proficiency had improved, with specific improvement in communication with patients, medical knowledge, and clinical reasoning, and that the student teams of 1^st^, 3^rd^ and 5^th^ year students performed at the level of junior doctors. The last one is especially important because competence at the level of junior doctors is expected from the students only after they finish their medical study.

In the regression analysis of the correlations between motivation and perceived competence improvement, we found the main intrinsic motivation outcome, the IMI interest subscale, was positively correlated with the students’ perceived improvement in CanMEDS competencies of medical expert and academic. The IMI-usefulness subscale which is important in self-regulation/internalization, seemed to have stronger correlations with perceived competence improvement. The latter could be expected, if a student would think his/her competences improved he/she would be more likely to consider the project useful (and thus have a higher IMI usefulness score, and vice versa). Even though expected, an earlier study in dentistry students showed no significant correlation between the IMI usefulness score and their competence (unfortunately, the IMI interest subscale was not used) [36]. An interesting finding in the regression analysis was the weak/absent correlation between the AMS intrinsic motivation and IMI subscales, this finding suggests the IMI has additional value next to the AMS. A possible explanation for this difference is the IMI measures the intrinsic motivation for a particular topic, and the AMS measures this for medical education in general.

The study had some limitations. The main limitation was the sample size and study design (no control group). Other limitations included a social desirability- and observer bias (participants could give socially desirable answers while filling out questionnaires, and faculty were not blinded while scoring students). Furthermore there was a selection bias, such that highly enthusiastic students might have applied and consequently have been selected, as discussed previously [9]. This selection might have influenced the (intrinsic) motivation, knowledge, and skills assessed during this study. The selection-bias was unavoidable, given the LC-SRC is a voluntary extracurricular activity with real patients. Therefore, the results regarding the clinical competence and (unchanged) high levels of motivation cannot be extrapolated to non-selected medical students in general. In spite of these limitations, we think that this exploratory pilot study contributes to our knowledge of motivation and learning in a student-run clinic, and helps in designing future studies. Such future research (with a control group) is needed to determine whether prolonged participation in a SRC setting improves competence and motivation for medical education.

## Conclusions

Students showed a high level of intrinsic motivation to participate in the LC-SRC and perceived an improvement in their competence. Furthermore their actual clinical competence was at junior doctor level in all CanMEDS competencies. We are of the opinion these competencies can be learned best in a setting similar to the future profession such as a LC-SRC, so that students are exposed to responsibility, real patient contact, and inter and intra disciplinary collaboration that is important to stimulate students’ intrinsic motivation. The LC-SRC offers an stimulating environment according to the theoretical framework based on the SDT. Together with the observed high levels of intrinsic motivation and the qualitative comments of the students in this study, this makes the LC-SRC an attractive place for learning.

## Additional file

Additional file 1: CanMEDS mini-CEX. “Supplement miniCEX CanMEDS competencies. Students’ CanMEDS competencies were evaluated by faculty from internal medicine using mini-CEX, to grade and provide feedback for student teams after each consultation. The mini-CEX form used is displayed in this supplementary file. (PDF 456 kb)

## Abbreviations

AMS: Academic Motivation Scale
IMI: Intrinsic Motivation Inventory
LC-SRC: Learner-centered student-run clinic
mini-CEX: Mini-Clinical Evaluation Exercise
SRC: Student-run clinic
VUmc: VU University Medical Center
