# Supplementary material for: Motivation and competence of participants in a learner-centered student-run clinic: an exploratory pilot study
Source: BMC Med Educ. 2017 Jan 25;17:23. doi: 10.1186/s12909-017-0856-9 (PMC5264437; doi:10.1186/s12909-017-0856-9)
Supplement: Additional file 1: — CanMEDS mini-CEX. “Supplement miniCEX CanMEDS competencies. Students’ CanMEDS competencies were evaluated by faculty from internal medicine using mini-CEX, to grade and provide feedback for student teams after each consultation. The mini-CEX form used is displayed in this supplementary file. (PDF 456 kb) [file 12909_2017_856_MOESM1_ESM.pdf]

Evaluation of CanMEDS competencies

After each consultation in the LC-SRC faculty from internal medicine evaluated the CanMEDS competencies for the team that performed the consultation. To evaluate the consultation the CanMEDS mini-CEX was used to grade and provide feedback. These CanMEDS mini-CEX were regularly used by faculty to evaluate clinical competence of students in their regular clerkships with a similar form. The seven CanMEDS competencies were scored on a 5-point Likert scale (compared to the level of a junior doctor, 3 meant achieving a junior doctor level, >3 better, <3 worse), see below.

Mini-Clinical Evaluation Exercise (Mini-CEX)

CANmeds competencies

Team, student names:		Assessor's name:					
		Clinical condition:					
Study years: B1 B2 B3 M1 M2 M3		Date of consultation:					
		**Not assess able**	**Under level of junior doctor**		**level of junior doctor**	**Beyond level of junior doctor**	
**Medical expert**	History taking & physical examination, distinguish main points and side issues, differential diagnosis & making a plan	N/A	1	2	3	4	5
**Communicator**	Patient contact (manners, respects) Giving information and advice	N/A	1	2	3	4	5
**Scholar**	Critically analyzing medical information sources and using it in a definite plan	N/A	1	2	3	4	5
**Professional**	Sense of responsibility, professional rules, to handle own emotions and difficult or impressive situations	N/A	1	2	3	4	5
**Collaborator**	Carrying and adapting medical information Respects for others → team player	N/A	1	2	3	4	5
**Organizer**	Structuring and planning, prioritizing, time management, attention to quality, appointments	N/A	1	2	3	4	5
**Health advocate**	Attention to safety of patients and policy of prevention	N/A	1	2	3	4	5
**Final assessment**			**1**	**2**	**3**	**4**	**5**
**Feedback:**							
